# Supplementary material for: Competing protein-protein interactions regulate binding of Hsp27 to its client protein tau
Source: Nat Commun. 2018 Nov 1;9:4563. doi: 10.1038/s41467-018-07012-4 (PMC6212398; doi:10.1038/s41467-018-07012-4)
Supplement: Supplementary file 1 — Supplementary Information [file 41467_2018_7012_MOESM1_ESM.pdf]

Supplementary Information

Competing Protein-Protein Interactions Regulate Binding of Hsp27 to Its Client Protein Tau

Freilich, R. et al. *Nature Communications*

Supplementary Table 1. List of primers used in this study.

Supplementary Figure 1. Schematic of all Hsp27 constructs used in this work.

Supplementary Figure 2. Representative ITC curves from Figures 1D and 4B. Raw data are shown.

Supplementary Figure 3. Hsp27 ACD has a weak affinity for K18 tau. (A) K18 tau was immobilized onto ELISA plates and saturated with biotin-labelled, human Hsc70, which shares binding sites with Hsp27 ACD in the MTBRs. Competition with unlabelled Hsc70 (control) or Hsp27 ACD was then carried out. Hsp27 ACD was estimated to have an IC50 > 100 µM. (B) Binding of Hsp27 ACD to K18 tau was not able to be measured by ITC, due to solubility limits. (C) NMR titrations (see Figure S5A) also suggested that the affinity of the interaction between K18 tau (D277-278) and Hsp287 ACD is weak. (D) NMR titration of VQIVYK peptide from tau into Hsp27 ACD (see Figure S5A) suggests a weak interaction. CSPs of Hsp27 ACD in the presence of 3.3-fold molar excess of PHF6* peptide (500 µM).

Supplementary Figure 4. Supplementary Figure 4. L157A ACD is deficient in BAG3 binding. Left, crystal structure (PDB: 4MJH) of Hsp27's IPV peptide bound to the b4/b8 groove of the ACD. Residue L157 is labelled. Right, interaction of Hsp27's ACD (WT and L157A) with BAG3 as measured by FCPIA.

Supplementary Figure 5. Hsp27's ACD binds two locations within K18 tau. (A) TROSY HSQC spectra of 15N WT K18 tau (red) in the presence of increasing concentrations of Hsp27 ACD. (B) Chemical shift perturbations of listed tau constructs in the presence of 2:1 molar ratio of ACD (120 µM). K18 DM is the double mutant.

Supplementary Figure 6. TROSY HSQC of full length Hsp27 with WT K18. CSPs of WT K18 in the presence of 3.6-fold molar excess of listed Hsp27.

Supplementary Figure 7. Sample aggregation curves of tau constructs with full-length Hsp27. (A) Four independent replicates of results shown in Figure 3A, using 0N4R tau. Concentrations are 10 µM. Error bars represent the spread of the four technical replicates. (B) Four independent replicates of the results shown in Figure 3A, using K18 tau. Concentrations are 10 µM. Error bars represent the spread of the four technical replicates. (C) At the end of the ThT assay, samples were centrifuged 10,000 xg for 10 min to separate the pellet (P) from the supernatant (S). The resulting samples were separated on SDS-PAGE and viewed by silver stain. Results are representative of experiments performed in at least quadruplicate. These results

suggest that the increase in ThT signal above the untreated tau samples (black in panels A and B) might be due, in part, to chaperone incorporation in a ThT-positive manner. These findings do not rule out the possibility that the chaperones also change the fibril sub-structure to enhance ThT signal.

Supplementary Figure 8. Oligomeric properties of Hsp27 constructs. (A) Differential scanning fluorimety melting curves of Hsp27 WT and 3D. Data points are the mean ± SEM of three technical replicates. (B) Negative stain EM images of WT (left) and GPG (right) Hsp27. Images are representative of a minimum of 12 random fields. Scale bar is 200 nm. (C) SEC-MALS trace of N-terminal deletion constructs (60 µM). (D) SDS-PAGE gel of Hsp27 and Hsp27 X.

Supplementary Table 1. Primers used in this study.

Primer Title	Sequence
L157A Forward	CAAGTTTCCTCCTCCGCGTCCCCTGAGGGCAC
L157A Reverse	GTGCCCTCAGGGGACGCGGAGGAGGAAACTTG
ΔNTD Forward LIC	TACTTCCAATCCAATGCAATGCAACTCAGCAGCG GGGTCTC
ΔNTD Reverse LIC	TTATCCACTTCCAATGTTACTTGGCGGCAGTCTCATCG

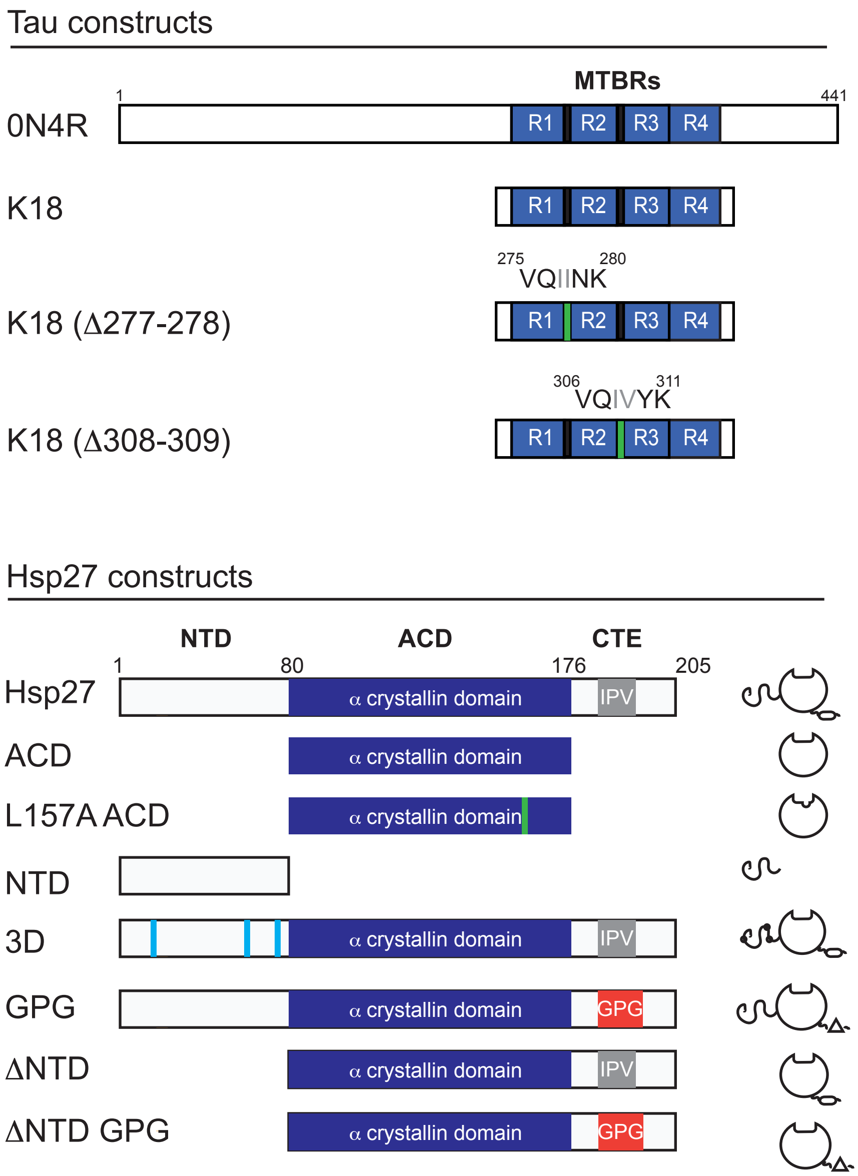

Supplemental Figure 1. Schematic of proteins used in this work. Mutations are indicated by green boxes.

A. Representative ITC curves from Figure 1D, alanine scan of IPV peptides.

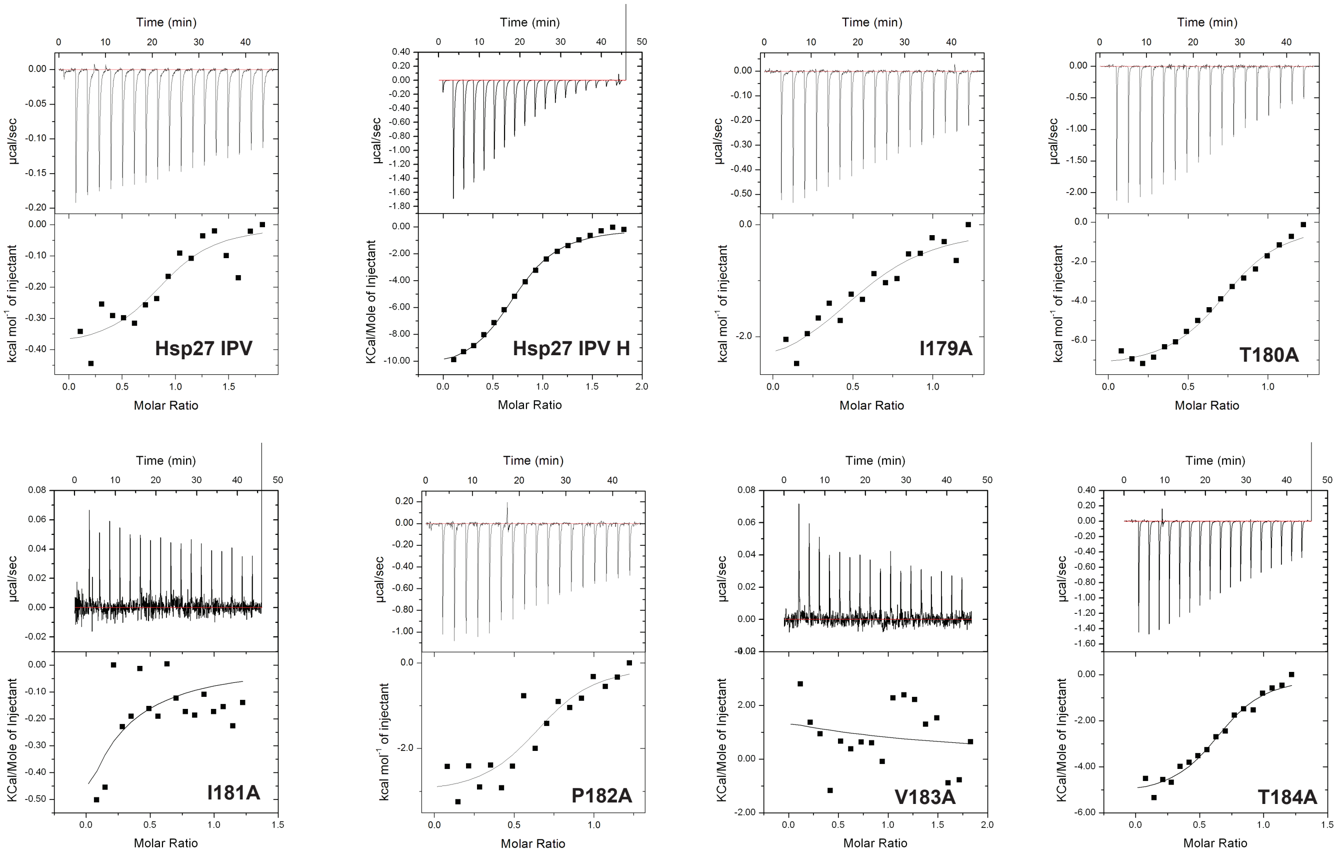

B. Representative ITC curves from Figure 4B, optimized IPV peptide with various Hsp27 constructs.

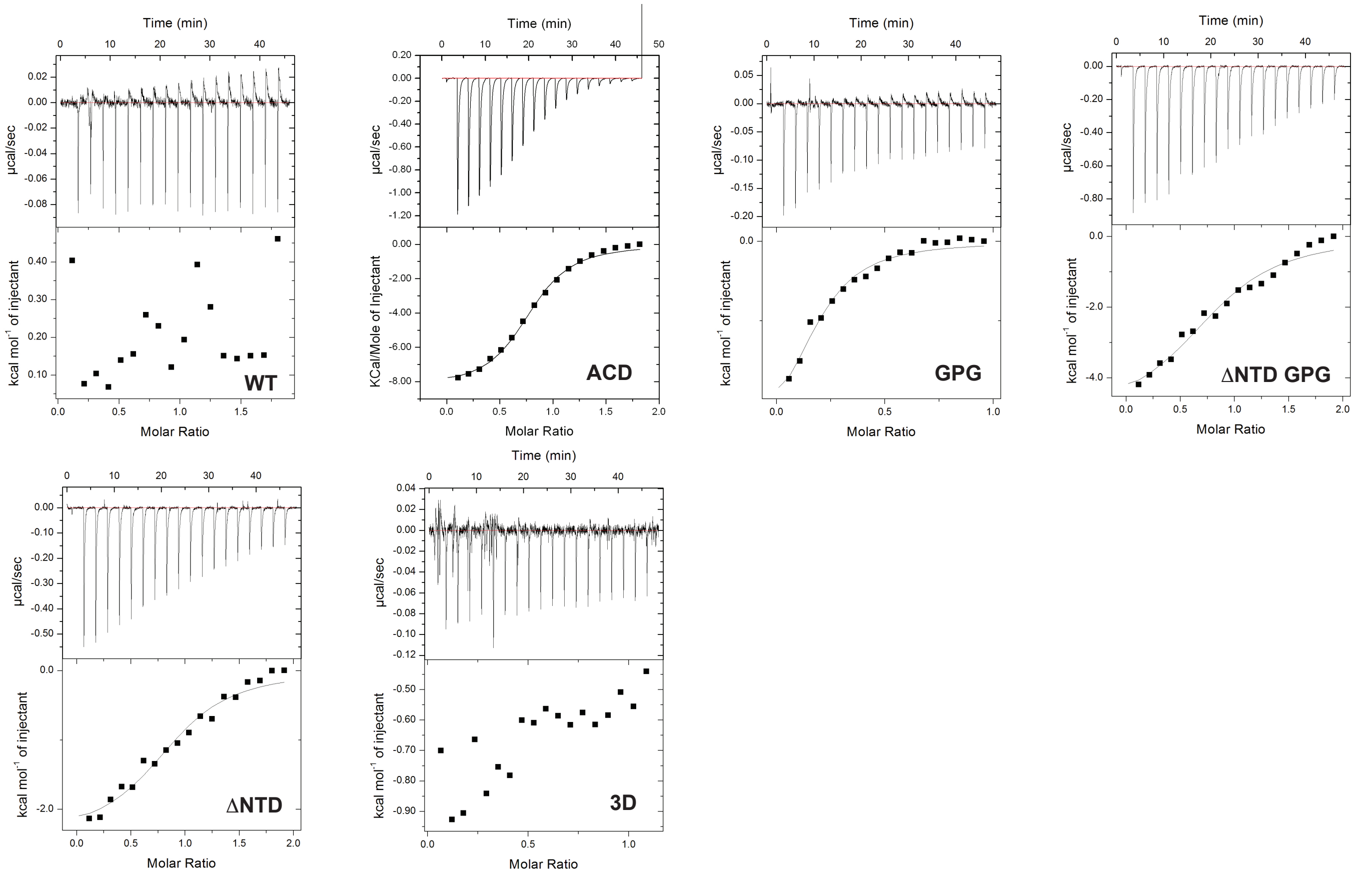

Supplementary Figure 2. Representative ITC curves (from Figures 1D and 4B). Raw data are shown.

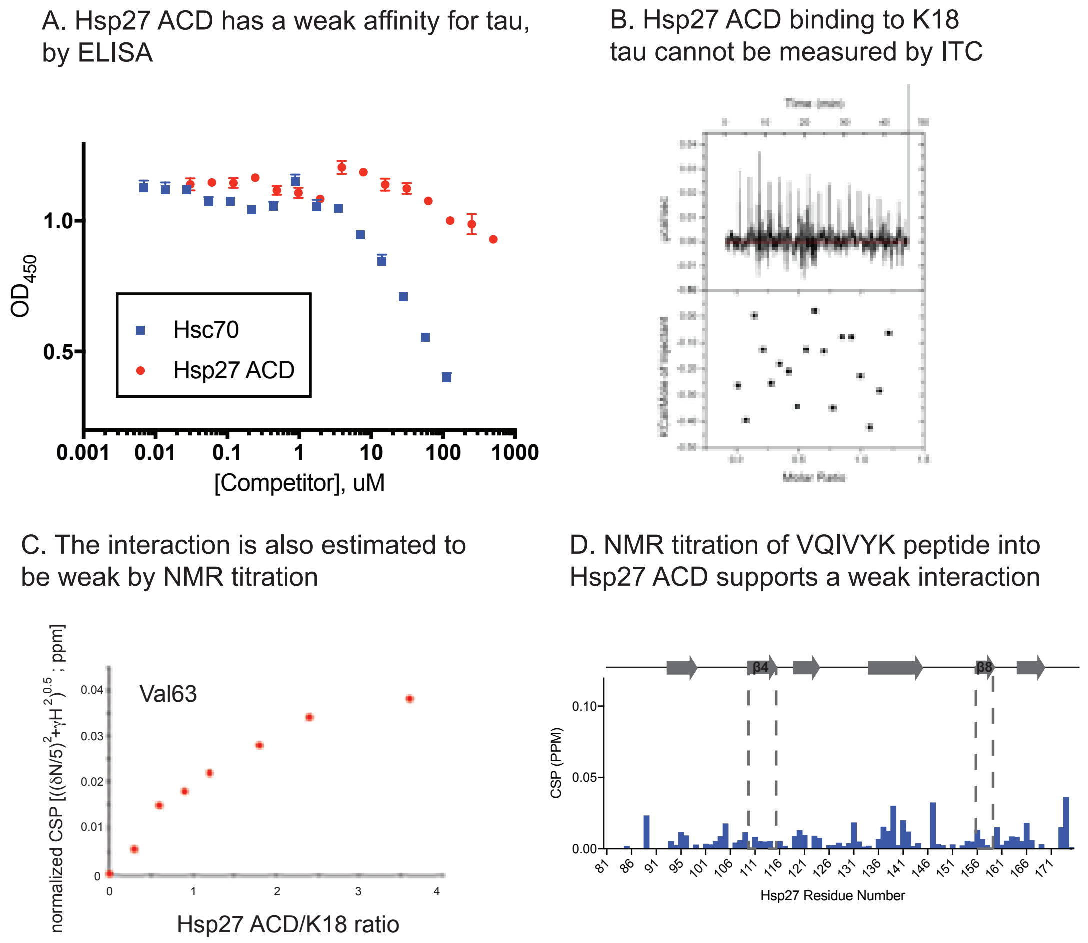

Supplementary Figure 3. Hsp27 ACD has a weak affinity for K18 tau. (A) K18 tau was immobilized onto ELISA plates and saturated with biotin-labelled, human Hsc70, which shares binding sites with Hsp27 ACD in the MTBRs. Competition with unlabelled Hsc70 (control) or Hsp27 ACD was then carried out. Hsp27 ACD was estimated to have an IC50 > 100 μM. (B) Binding of Hsp27 ACD to K18 tau was not able to be measured by ITC, due to solubility limits. (C) NMR titrations (see Figure S5A) also suggested that the affinity of the interaction between K18 tau (Δ277-278) and Hsp287 ACD is weak. (D) NMR titration of VQIVYK peptide from tau into Hsp27 ACD (see Figure S5A) suggests a weak interaction. CSPs of Hsp27 ACD in the presence of 3.3-fold molar excess of PHF6* peptide (500 μM).

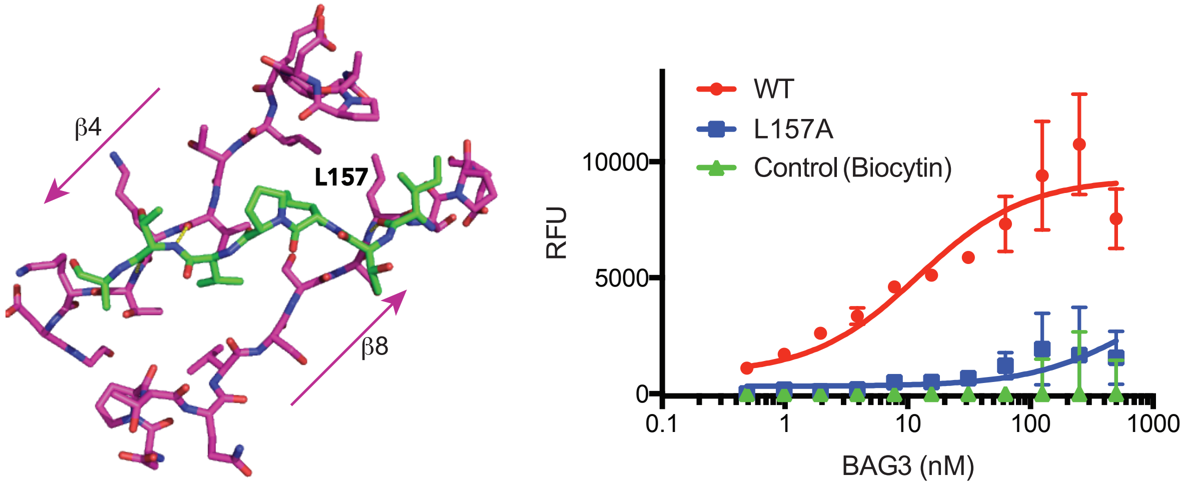

Supplementary Figure 4. L157A ACD is deficient in BAG3 binding. Left, crystal structure (PDB: 4MJH) of Hsp27's IPV peptide bound to the β4/β8 groove of the ACD. Residue L157 is labelled. Right, interaction of Hsp27's ACD (WT and L157A) with BAG3 as measured by FCPIA.

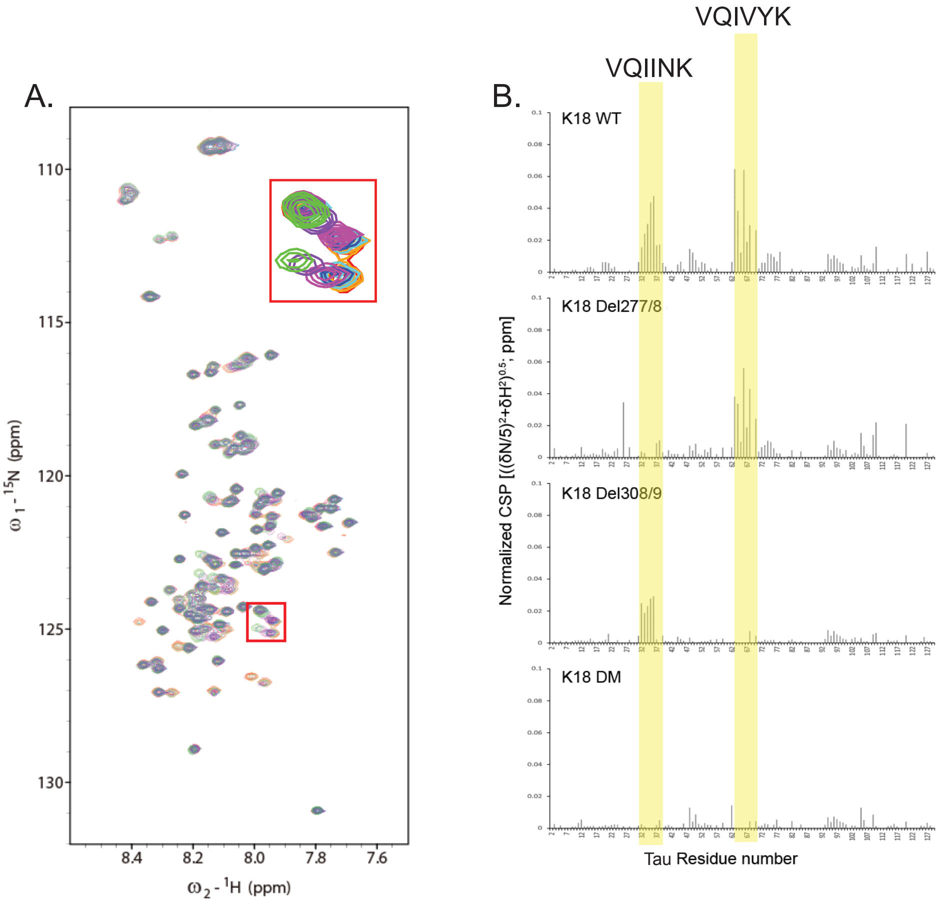

Supplementary Figure 5. Hsp27's ACD binds two locations within K18 tau. (A) TROSY HSQC spectra of 15N WT K18 tau (red) in the presence of increasing concentrations of Hsp27 ACD. (B) Chemical shift perturbations of listed tau constructs in the presence of 2:1 molar ratio of ACD (120 μM). K18 DM is the double mutant.

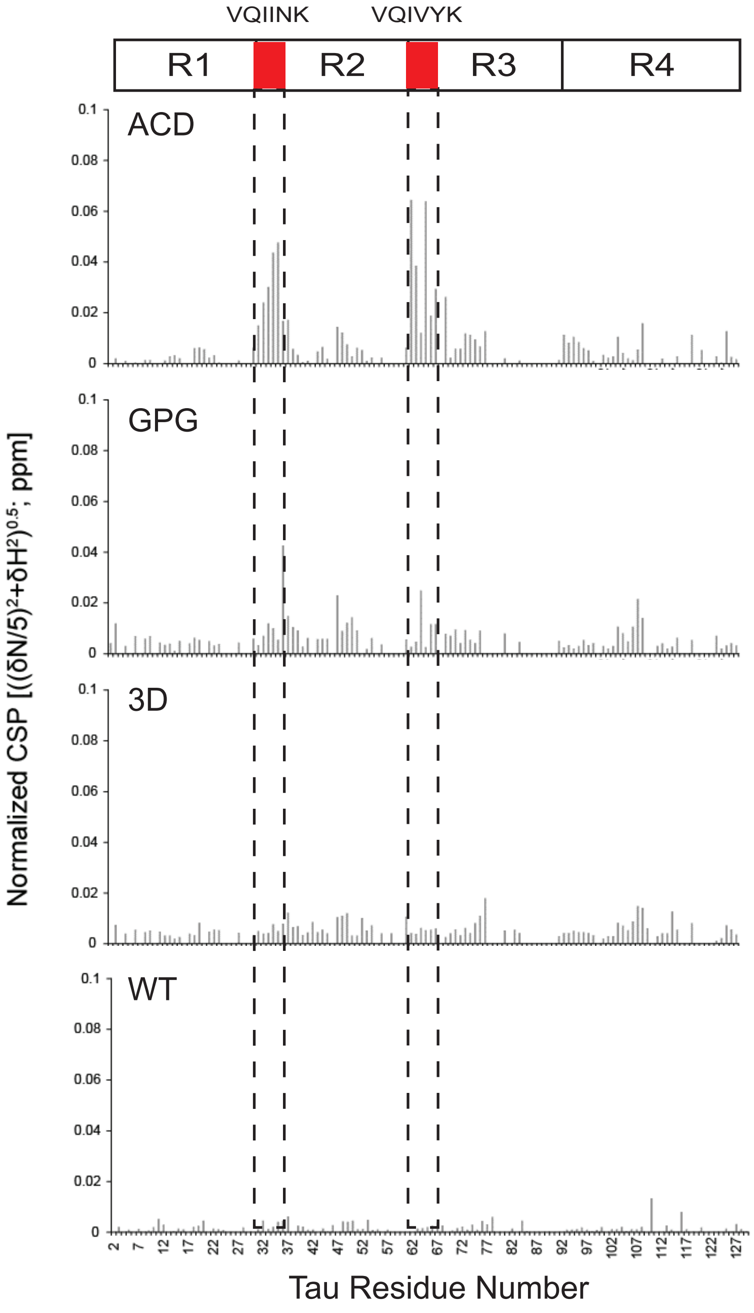

Supplementary Figure 6. TROSY HSQC of full length Hsp27 with WT K18. CSPs of WT K18 in the presence of 3.6-fold molar excess of listed Hsp27.

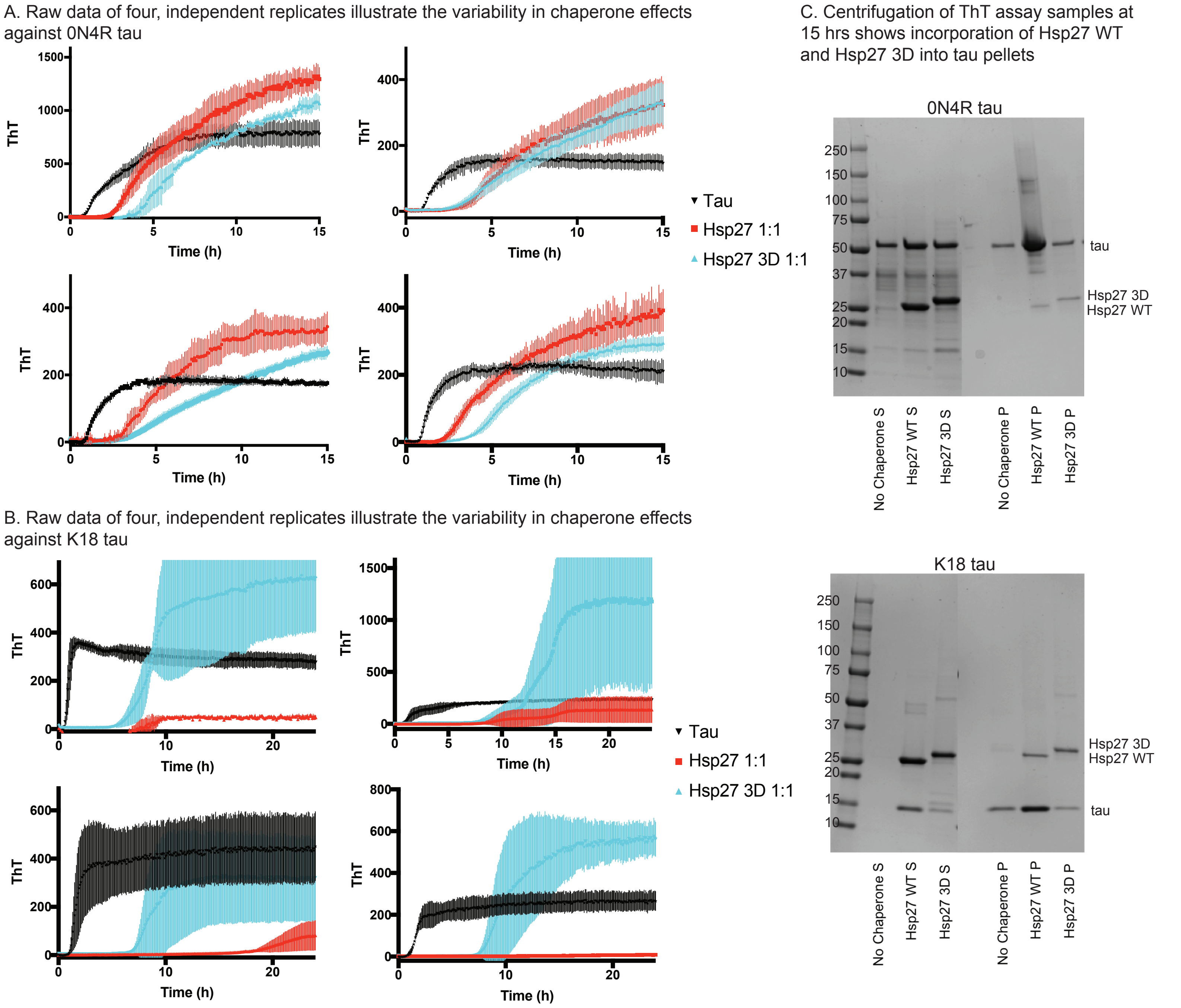

Supplementary Figure 7. Sample aggregation curves of tau constructs with full-length Hsp27. (A) Four independent replicates of results shown in Figure 3A, using 0N4R tau. Concentrations are 10 μM. Error bars represent the spread of the four technical replicates. (B) Four independent replicates of the results shown in Figure 3A, using K18 tau. Concentrations are 10 μM. Error bars represent the spread of the four technical replicates. (C) At the end of the ThT assay, samples were centrifuged 10,000 xg for 10 min to separate the pellet (P) from the supernatant (S). The resulting samples were separated on SDS-PAGE and viewed by silver stain. Results are representative of experiments performed in at least quadruplicate. These results suggest that the increase in ThT signal above the untreated tau samples (black in panels A and B) might be due, in part, to chaperone incorporation in a ThT-positive manner. These findings do not rule out the possibility that the chaperones also change the fibril sub-structure to enhance ThT signal.

A. T_M of Hsp27 WT and 3D

	WT	3D
T_M (°C)	72.2 ± 1.0	72.2 ± 0.85

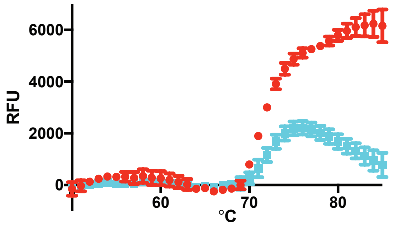

B. Superstructure of Hsp27 WT and GPG

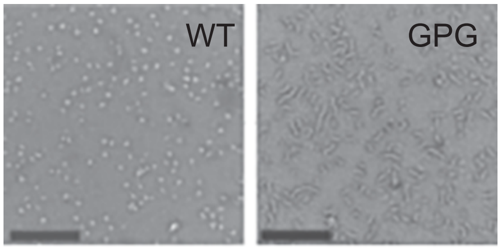

C. Oligomeric properties of NTD-deletion constructs

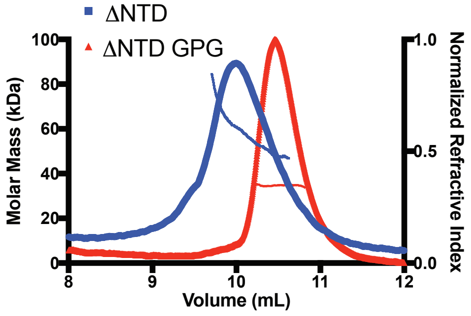

Hsp27	MW (kDa)	Range (kDa)	Subunits
ΔNTD	58	85-47	3-5
ΔNTD GPG	34	34-33	2

D. SDS-PAGE gel of Hsp27 and Hsp27 X

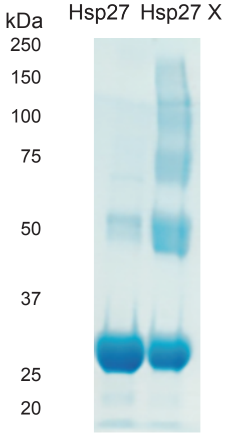

Supplementary Figure 8. Oligomeric properties of Hsp27 constructs. (A) Differential scanning fluorimety melting curves of Hsp27 WT and 3D. Data points are the mean ± SEM of three technical replicates. (B) Negative stain EM images of WT (left) and GPG (right) Hsp27. Images are representative of a minimum of 12 random fields. Scale bar is 200 nm. (C) SEC-MALS trace of N-terminal deletion constructs (60 μM). (D) SDS-PAGE gel of Hsp27 and Hsp27 X.
